# A Study on Imaging Risk Factors for Hip Osteoarthritis

**DOI:** 10.1111/os.14173

**Published:** 2024-08-21

**Authors:** Fan Wang, Peng Yuan, Yixin Gong, Guohao Zhang, Pengcui Li, Qiang Jiao

**Affiliations:** ^1^ Department of Orthopedics The Second Hospital of Shanxi Medical University Taiyuan China; ^2^ Department of Orthopedics The Second Hospital of Shanxi Medical University, Shanxi Key Laboratory of Bone and Soft Tissue Injury Repair Taiyuan China

**Keywords:** Hip Osteoarthritis, Imaging, Morphological Feature, Pathogenesis

## Abstract

**Objective:**

Due to low prevalence and few studies, the morphologic risk factors for hip osteoarthritis (HOA) in Chinese population remain unknown. The purpose of this study was to investigate the relationship between 10 radiographic parameters measured via anteroposterior pelvic X‐ray radiography and HOA in Chinese population.

**Methods:**

Thirty‐three patients who required total hip arthroplasty for unilateral HOA (2017–2022) and 132 healthy individuals were selected for this case–control study. We measured 10 radiological parameters via anteroposterior pelvic X‐ray radiography, which were sharp angle, center edge angle, sourcil angle, neck shaft angle, α angle, pelvic height, pelvic width, femoral head diameter, femoral neck width, and ratio of the femoral head diameter to the femoral neck width. After measurements were obtained, logistic regression analysis was utilized to calculate the adjusted odds ratios (ORs) for confounding variables such as age, sex, and body mass index (BMI). Receiver operating characteristic (ROC) curves were utilized to determine the proportional risk contribution (PRC) of each radiographic factor.

**Results:**

After adjustment for confounding factors, individuals with a larger sourcil angle (SA) (OR = 4.89, 95% CI 1.66–14.42, *p* = 0.004), larger α angle (OR = 4.14, 95% CI 1.53–11.23, *p* = 0.005), and wider femoral neck (OR = 5.27, 95% CI 1.50–18.51, *p* = 0.01) were found to have a greater risk of developing HOA. Among all radiographic parameters, the SA demonstrated the greatest risk contribution (PRC = 13.695%).

**Conclusions:**

Radiographic parameters correlate with the incidence of HOA. The SA is probably the most powerful of all the parameters related to HOA.

## Graphical abstract

The purpose of this study is to investigate the relationship between 10 radiographic parameters measured via anteroposterior pelvic X‐ray radiography and hip osteoarthritis (HOA). We found that in patients without significant hip dysplasia, a larger SA, wider femoral neck, nonspherical femoral head, pistol grip deformity, and smaller PW were risk factors for HOA.

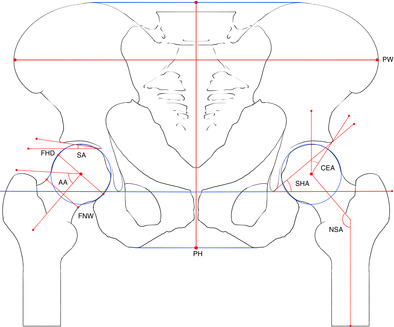

## Introduction

Hip osteoarthritis (HOA) is a prevalent degenerative and disabling joint disorder that poses a substantial burden on individuals, nations, and societies.^1^ The pathogenesis of HOA is complex and encompasses various factors, including age, sex, obesity, genetics, and joint injuries.^2^ Over the years, many of studies have highlighted that local morphological abnormalities play a crucial role in the development of HOA,^3^ and excessive or inadequate coverage of the acetabulum, a shallow acetabulum, cam deformity of the femoral head, and periarticular bone density around the hip joint may have a more significant impact on the incidence of HOA.^4^, ^5^, ^6^ Thus, in recent years, myriad studies examining the relationship between HOA and radiographic parameters have emerged. For instance, Doherty et al. identified a nonspherical femoral head shape (pistol grip deformity) and a large neck shaft angle (NSA) as risk factors for HOA.^7^ Conversely, a recent report revealed no statistically significant difference in the NSA between the patient and control groups.^5^ Iidaka^8^ revealed that acetabular dysplasia in the Japanese population is a risk factor for the onset and progression of HOA. Abdulrahim et al.^9^ identified 14 measurement indicators on anteroposterior pelvic radiographs associated with HOA, among which an excessively large sourcil angle (SA) increased the risk of developing HOA nearly sevenfold in healthy individuals. These studies suggest a close association between local hip morphological variations and the onset of HOA.

However, to date, the primary focus of these studies has been on populations in Europe and the Americas, with relatively limited research on radiographic risk factors for HOA in the Chinese population. A study targeting HOA in the Chinese population indicated that the femoral anteversion angle is a risk factor for HOA; however, the study population included only patients with acetabular dysplasia.^10^ In fact, from a regional perspective, the prevalence of HOA varies between Asia and Europe. According to reports, the prevalence of HOA in the European population is approximately 6–11%, while in the Chinese population, it is around 0.6%.^11^ This significant variation is largely associated with ethnic differences.^12^ Therefore, studies exploring morphological risk factors for HOA are necessary, as they can help elucidate the pathogenesis of HOA. The aim of the present study was to investigate a more comprehensive set of morphological risk factors for HOA that had been demonstrated in other ethnographic studies in the Chinese population. We hypothesized that the healthy hip joint in these patients represents the pre‐disease state, which has already been supported in several large case–control studies.^7^, ^9^, ^13^ We sought to determine whether the morphological changes in the hip joint identified in other populations are still meaningful and to calculate the proportional risk contribution (PRC) of these radiographic parameters.

Therefore, the aims of this study were as follows: (1) To validate the bilateral symmetry of the hip joints by measuring radiological parameters around the hip joint. This approach aids in exploring whether the healthy side in unilateral HOA patients can function as a morphological reference prior to disease onset on the affected side. (2) To calculate the odds ratios (ORs) to investigate whether these characteristics serve as risk or protective factors for HOA.

## Methods

### Patients and Control Participants

**Figure 1:**
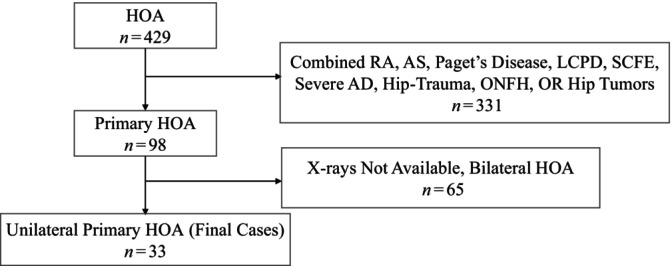
Flowchart for screening patients. AD, acetabular dysplasia; AS, ankylosing spondylitis; HOA, hip osteoarthritis; LCPD, Legg–Calvé–Perthes disease; ONFH, osteonecrosis of the femoral head; RA, rheumatoid arthritis; SCFE, slipped capital femoral epiphysis.

Patients with unilateral HOA (from 2017 to 2022) were selected for inclusion in the study. All patients had been referred to the hospital with symptomatic, clinically severe hip OA, and had undergone total hip arthroplasty (THA). Subjects were excluded from the study if they had any of the following comorbidities: (i) major arthropathy (rheumatoid arthritis, ankylosing spondylitis, Paget's disease of bone affecting the pelvis or femur); (ii) overt childhood hip disease (Legg–Calvé–Perthes disease, slipped capital femoral epiphysis, severe acetabular dysplasia); (iii) THA due to trauma or osteonecrosis of the femoral head; or (iv) a terminal illness. Finally, 33 patients with unilateral HOA with complete imaging films were included in the study. A flowchart can be found in Figure 1. For the control group, we recruited a group of people aged from 45 to 80 years from the physical examination department of our hospital and obtained supine anteroposterior pelvic radiographs. Individuals who had no radiographic evidence of hip OA on their pelvic films, exhibited no hip pain, and had no surgical trauma history were selected as control participants for this study, totaling 218 individuals. In order to get a ratio of 4:1 between the control and patient groups, we used the random number method to select 132 individuals as controls for this study. HOA was indicated radiographically when minimum joint space width (mJSW) was ≤2.5 mm.^14^ Subjects in the patient group with mJSW ≤2.5 mm in either hip were classified as having HOA. Subjects with mJSW >2.5 mm in both hips were retained as control participants. Demographic data such as age, height, weight, and body mass index (BMI) were collected. All participants in the study were Chinese.

### Radiographic Assessment

**Figure 2:**
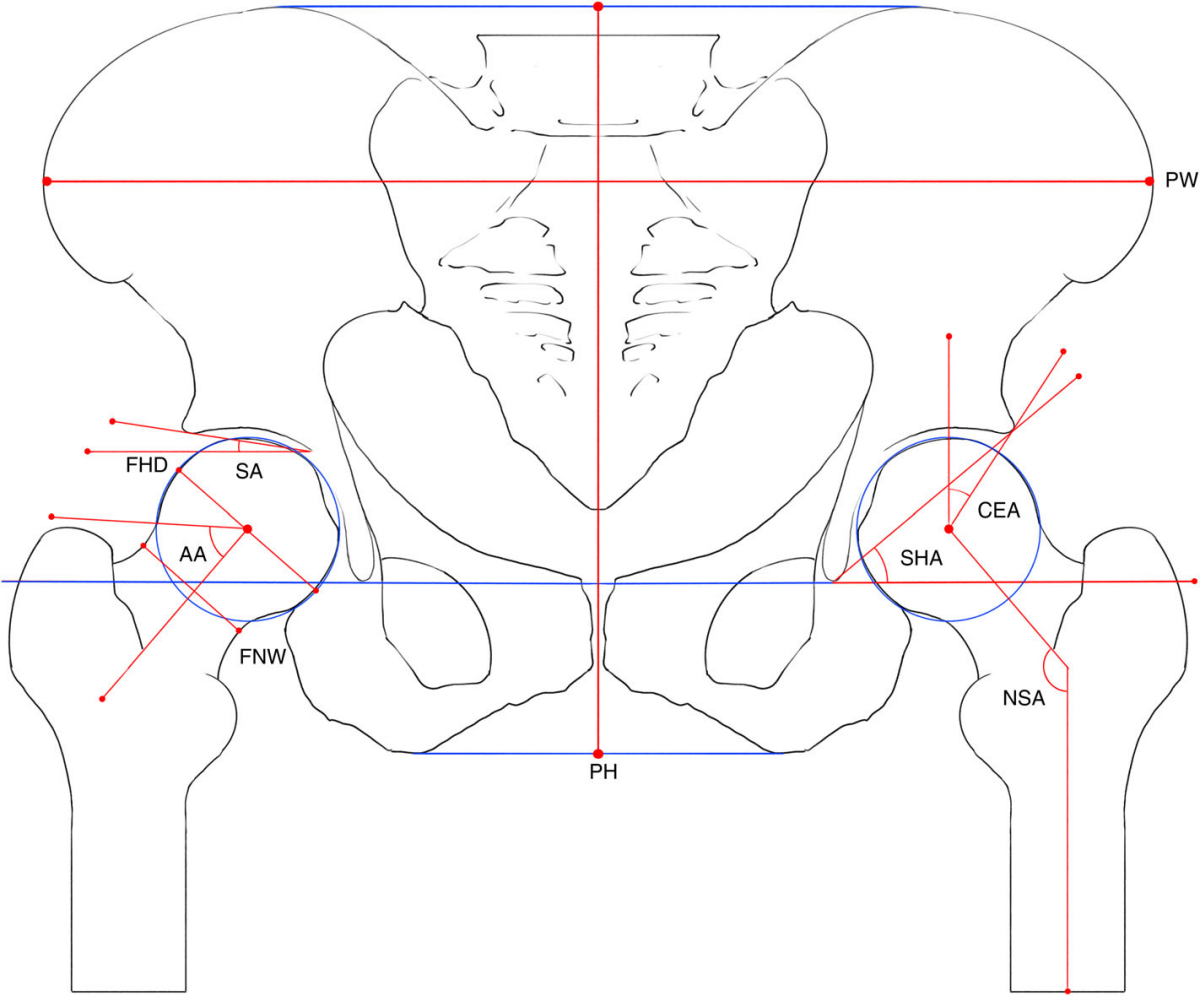
Diagram showing the radiographic parameters of the hip and pelvic bones. 20 × 16 mm (9449 × 7559 DPI). AA, α angle; CEA, center edge angle; FHD, femoral head diameter; FHNR, the ratio of femoral head diameter divided by femoral neck width; FNW, femoral neck width; NSA, neck shaft angle; OA, osteoarthritis; PH, pelvic height; PW, pelvic width; SA, sourcil angle, SHA, sharp angle.

**Figure 3:**
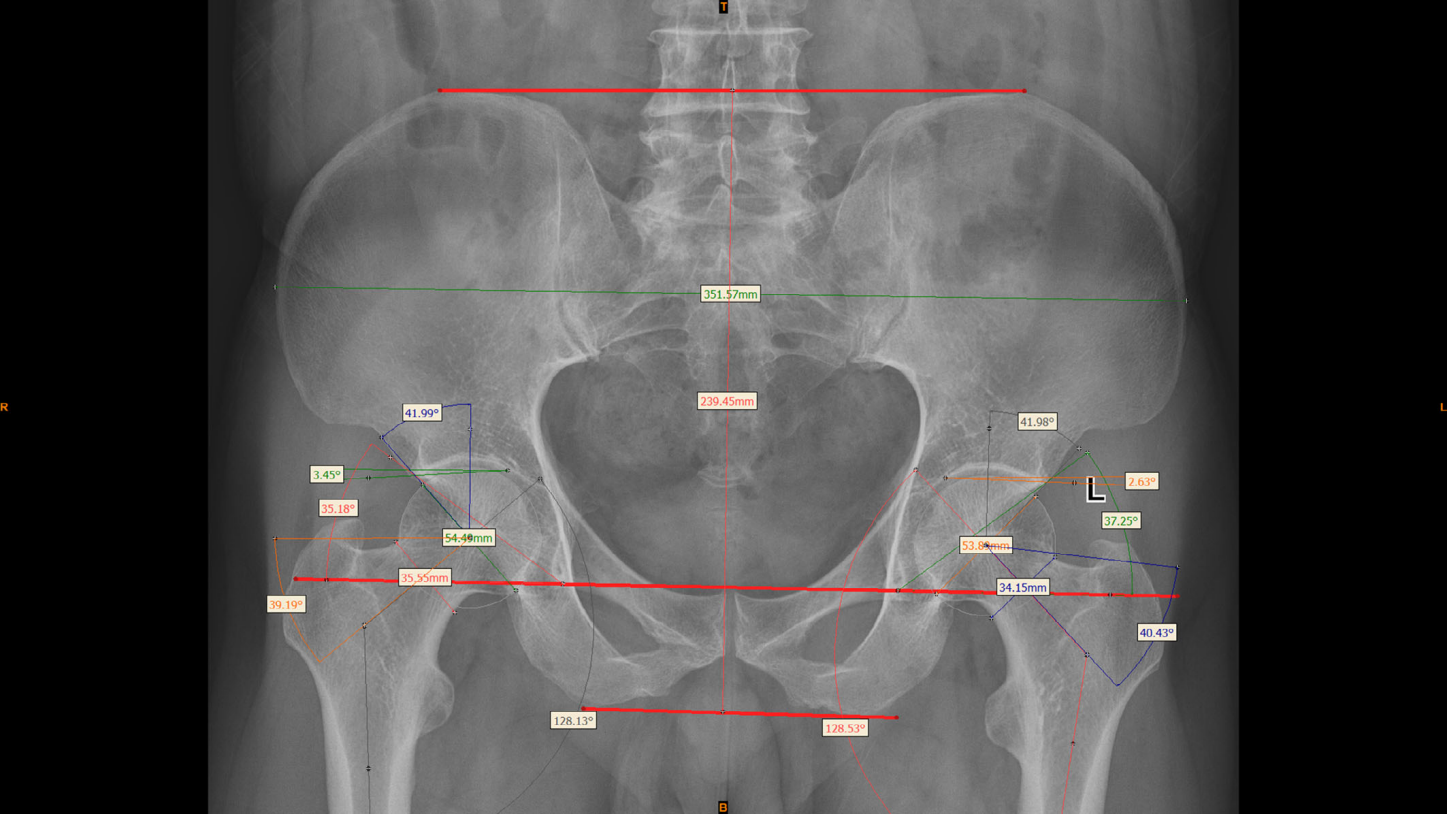
A screenshot of Mimics medical software (control group).

All participants underwent non‐weight‐bearing anteroposterior pelvic X‐ray examinations. During the procedure, patients were supine with both feet internally rotated 10°, with the toes of both feet in contact. Using Mimics Medical 21.0 software^15^ on a computer, we annotated and measured 10 radiological parameters. Two physicians who were blinded to patient name and age independently obtained measurements in the patient group and the control group according to the following method. The X‐ray images were opened with Mimics Medical software; a line was drawn between the bilateral pelvic teardrops to serve as the horizontal line of the pelvis, and the concentric circle method was used to find the most suitable circle for the femoral head. Then, the software's measurement tool was used to measure the following 10 radiological parameters: the Sharp angle (SHA), CEA, SA, NSA, α angle (AA), pelvic height (PH), pelvic width (PW), femoral head diameter (FHD), femoral neck width (FNW), and ratio of the femoral head diameter to the femoral neck width (FHNR).^7^ The features that were measured are illustrated and described in Figure 2 and Table 1. A screenshot of the software can be found in Figure 3. Notably, the AA was measured on the anteroposterior view. For patients with unilateral HOA, these radiographic features were measured on the healthy side. In the healthy population, features were measured on both sides.

**Table 1:** Descriptions of the radiographic parameters of the pelvis and hip joint.

Radiographic parameters	Descriptions
Sharp angle	The angle formed by the line connecting both teardrops and the line from the upper outer edge of the pelvic acetabulum to the ipsilateral teardrop.
Center edge angle	The angle formed by the perpendicular line from the center of the femoral head to the teardrop line and the line connecting the center of the femoral head to the upper outer edge of the acetabulum.
Sourcil angle	The angle between the line connecting the inner and outer sides of the acetabular sclerosis edge and the teardrop line.
Neck shaft angle	The angle between the femoral shaft axis and the femoral neck axis.
Α angle	The angle between the femoral neck axis and the line connecting the center of the femoral head to the point where the femoral head loses its spherical shape.
Pelvic height	The greatest height of the pelvic bone at the center of the pelvis on the radiograph.
Pelvic width	The widest diameter of the pelvic bone on the radiograph.
Femoral head diameter	The maximum diameter perpendicular to the femoral neck axis through the center of the femoral head.
Femoral neck width	The distance perpendicular to the femoral neck axis at the narrowest point of the femoral neck.
Femoral head to femoral neck ratio	The ratio of femoral head diameter divided by the femoral neck width.

*Note*: Table 1 shows the definitions of the 10 measured radiographic parameters of the pelvis and hip joint.

### Statistical Analysis

We used the intraclass correlation coefficient (ICC) to validate the consistency of measurements made by two doctors and paired t tests and nonparametric tests to detect the symmetry of radiological examination indicators on the left and right sides in the control group. Pearson's correlation coefficient was used to check the correlation between measurements. We divided continuous data into three groups, namely, T1, T2, and T3, using tertiles, and different control participants were selected according to whether the independent and dependent variables showed a linear or “U‐shaped” relationship. A logistic regression model was used to calculate the ORs and 95% confidence intervals (CIs) for each radiological indicator against HOA, adjusting for confounding factors such as age, sex, and BMI.

The reliability of the prediction model was estimated using receiver operating characteristic (ROC) curves, and the area under the ROC curve (AUC) was calculated. The PRC of each risk factor was calculated via a formula.^16^ First, we established a ROC curve for each risk factor and calculated the AUC, defined as AUC_f_, with the intent of representing a full risk model incorporating all available risk factors. The comprehensive risk model included confirmed risk factors, such as age, sex, weight, height, and BMI, and our measured radiological indicators. Then, we calculated the AUC (AUC_p_) after removing individually studied risk factors. Finally, we calculated the PRC using the following formula: PRC = (AUC_f_ − AUC_p_)/(AUC_f_ − 0.5), where 0.5 is the AUC under the diagonal line of the ROC curve index.^9^ Data analyses were performed using SPSS Statistics 27 and R, with significant differences defined at *p* < 0.05.

## Results

### Characteristics of the Study Participants

The general characteristics of the participants are shown in Table 2. A total of 165 individuals participated in the study; among them, 33 had unilateral HOA and composed the patient group, and 132 were healthy individuals above 45 years of age and composed the control group. The proportion of females was 69.7% in the patient group and 66.7% in the control group. In the patient group, 48.5% of patients had right‐sided HOA, while 51.5% had left‐sided HOA. Except for age in the control group, which did not follow a normal distribution, the other indicators were normally distributed. According to t tests and nonparametric tests, there was no significant difference between groups in terms of age, sex, height, weight, or BMI.

**Table 2:** The groups’ characteristics.

	Unilateral OA group (*n* = 33)	Non‐OA control participant (*n* = 132)
Age (years) mean ± SD or age (years) Median (Q1, Q3)	64.36 ± 7.22	65 (59, 69.75)
Women (%)	69.7	66.7
BMI (kg/m^2^) mean ± SD	24.80 ± 3.15	24.54 ± 2.67
Height (cm) mean ± SD	160.85 ± 5.8	163.13 ± 7.48
Weight (kg) mean ± SD	64.36 ± 7.2	65.43 ± 9.15

*Note*: Table 2 shows the general characteristics of the participants.

Abbreviations: BMI, body mass index; OA, osteoarthritis; Q1, first quartile; Q2, second quartile; Q3, third quartile; SD, standard deviation.

### Repeatability of Measurements

The ICCs of the nine radiographic parameters, as assessed by two physicians, ranged from 0.896 to 0.988, indicating a high level of consistency in the measured indices (SHA, 0.946; CEA, 0.954; SA, 0.979; NSA, 0.966; AA, 0.978; FHD, 0.988; FNW, 0.966; PH, 0.896; PW, 0.980).

### Symmetry in Healthy Control participants

In the healthy control group, the SA, AA, and FNW did not follow a normal distribution and were assessed for symmetry using nonparametric tests., while paired *t* tests were used for normally distributed variables. As shown by the statistical tests, apart from the AA, the bilateral data for the remaining seven indicators (since each person has only one PH and PW, symmetry was not tested) were significantly symmetrical.

To explore whether the asymmetry of the AA was due to sampling errors from a small sample size, we used bilateral AA data from the 218 initially recruited healthy individuals for statistical analysis. We found no significant difference between the left and right sides (*p* = 0.245). The detailed data can be found in Table 3.

**Table 3:** Results of paired t tests and nonparametric tests performed between the left and right sides to assess symmetry.

Radiographic parameters	Mean (SD)/Median (Q1, Q3)		Paired *t*‐tests/non‐parametric tests
	Left	Right	*p*
CEA	34.83 (5.98)	34.62 (5.53)	0.484
SHA	37.58 (3.86)	37.62 (3.44)	0.837
NSA	128.33 (5.02)	128.36 (4.66)	0.911
SA	3.69 (0.72, 7.08)	4.41 (0.82, 6.33)	0.900
FHD	53.14 (3.93)	53.30 (3.83)	0.120
FNW	35.33 (33.45, 37.66)	35.15 (33.36, 37.78)	0.167
FHNR	1.50 (0.79)	1.50 (0.81)	0.487
AA (*n* = 132)	43.01 (39.59, 46.80)	42.16 (39.64, 45.21)	0.003*
AA (*n* = 218)	42.71 (39.35, 46.47)	42.19 (39.86, 45.30)	0.245

*Note*: Table 3 demonstrates that the eight features were significantly symmetrical.

Abbreviations: AA, α angle; CEA, center edge angle; FHD, femoral head diameter; FHNR, the ratio of femoral head diameter divided by femoral neck width; FNW, femoral neck width; NSA, neck shaft angle; Q1, first quartile; Q2, second quartile; Q3, third quartile; SA, sourcil angle; SHA, sharp angle; SD, standard deviation.

* *p* < 0.01.

### Risk of HOA

**Figure 4:**
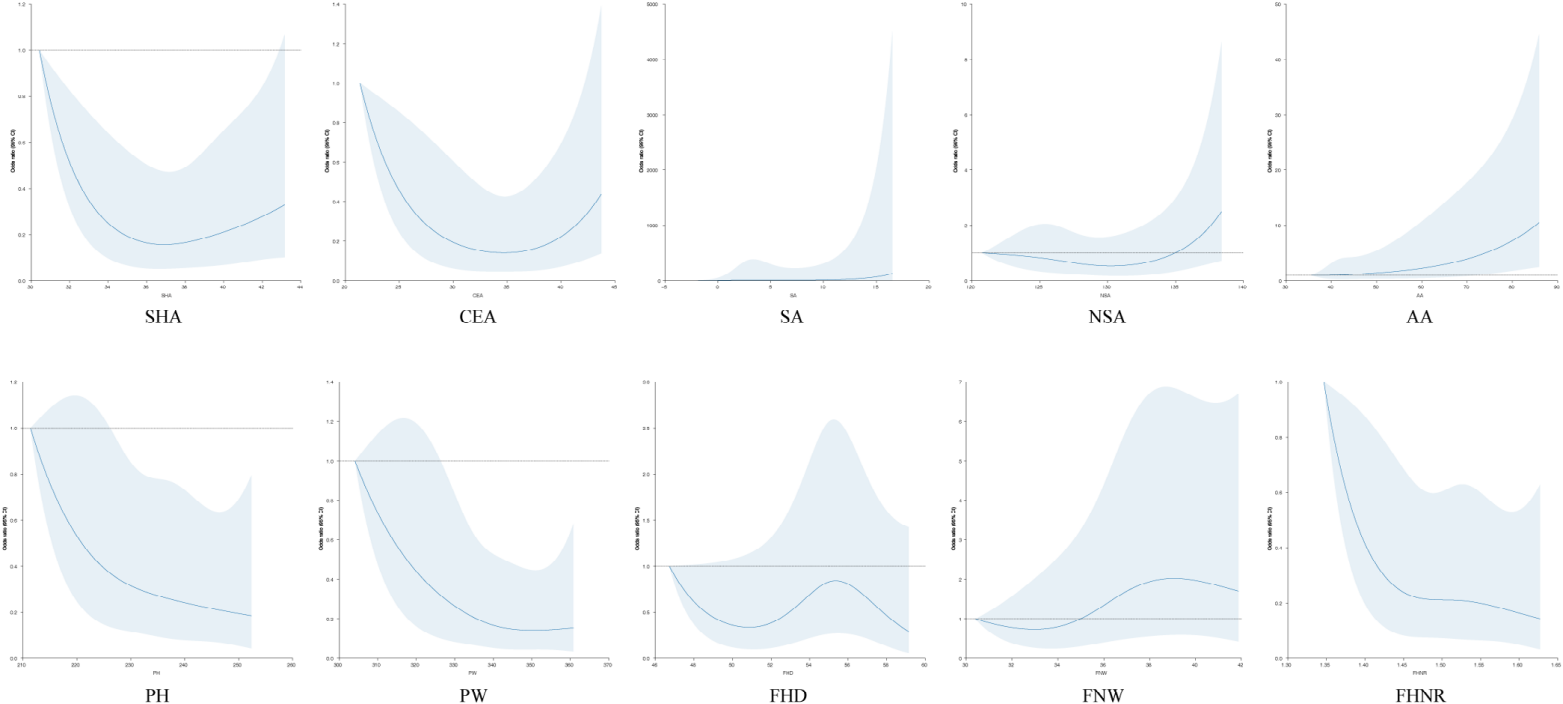
Diagram showing the graph of restricted cubic splines. The SHA, NSA, and FHD presented a “U‐shaped” relationship (both higher and lower values associating with increased/decreased risk) with the disease, while all other indicators showed a linear relationship. AA, α angle; CEA, center edge angle; FHD, femoral head diameter; FHNR, the ratio of femoral head diameter divided by femoral neck width; FNW, femoral neck width; NSA, neck shaft angle; OA, osteoarthritis; PH, pelvic height; PW, pelvic width; SA, sourcil angle; SHA, sharp angle.

Through logistic regression analysis, the OR of HOA for each radiological indicator was obtained, as shown in Table 4. If the radiographic indicator and the incidence of HOA showed a linear relationship, the T1 group was used as a control. For “U‐shaped” relationships, the T2 group was used as a control. Details are indicated in Figure 4. The SHA, NSA, and FHD presented a “U‐shaped” relationship with HOA, while all other indicators showed a linear relationship. Through statistical analysis, we found that the SA (T3, OR: 4.87, 95% CI: 1.66–14.30 *p* = 0.004), AA (T3, OR: 3.36 95% CI: 1.33–8.50 *p* = 0.005), and FNW (T3 OR: 2.86 95% CI: 1.01–8.12 *p* = 0.049) were risk factors for HOA, while a larger PH (T3 OR: 0.33 95% CI: 0.12–0.92 *p* = 0.034), PW (T3 OR: 0.27 95% CI: 0.10–0.76 *p* = 0.013), and FHNR (T2 OR: 0.35 95% CI: 0.14–0.89 *p* = 0.028; T3 OR: 0.30 95% CI: 0.11–0.79 *p* = 0.015) were protective factors against HOA. After adjusting for age, sex, and BMI, the difference in the OR for a larger PH was no longer significant (T3 OR: 0.31 95% CI: 0.08–1.21 *p* = 0.092), but other indicators retained their statistical significance. For the SHA, the risk of developing HOA among those with a smaller SHA had an OR of 2.52 (95% CI: 0.94–6.76), but the difference was not statistically significant (*p* = 0.066).

**Table 4:** Radiographic parameters and their associations with HOA.

	Frequency (%)		OR (95% CI)		*p*	
	OA group	Control participants	Crude	Adjusted	*p* (Crude)	*p* (Adjusted)
SHA						
T1	15 (45.45%)	40 (30.30%)	2.25 (0.87 ~ 5.85)	2.52 (0.94 ~ 6.76)	0.096	0.066
T2	8 (24.24%)	48 (36.36%)	*r*	*r*		
T3	10 (30.30%)	44 (33.33%)	1.36 (0.49 ~ 3.77)	1.42 (0.51 ~ 3.95)	0.55	0.508
CEA						
T1	12 (36.36%)	43 (32.58%)	1.00 (0.41 ~ 2.47)	0.93 (0.37 ~ 2.37)	1	0.881
T2	12 (36.36%)	43 (32.58%)	*r*	*r*		
T3	9 (27.27%)	46 (34.85%)	0.70 (0.27 ~ 1.83)	0.67 (0.25 ~ 1.81)	0.468	0.433
SA						
T1	5 (15.15%)	50 (37.88%)	*r*	*r*		
T2	10 (30.30%)	45 (34.09%)	2.22 (0.71 ~ 6.70)	2.13 (0.66 ~ 6.83)	0.172	0.204
T3	18 (54.55%)	37 (28.03%)	4.87 (1.66 ~ 14.30)**	4.89 (1.66 ~ 14.42)**	0.004	0.004
NSA						
T1	10 (30.30%)	45 (34.09%)	*r*	*r*		
T2	10 (30.30%)	45 (34.09%)	1.00 (0.38 ~ 2.64)	0.97 (0.36 ~ 2.57)	1	0.948
T3	13 (39.39%)	42 (31.82%)	1.39 (0.55 ~ 3.51)	1.34 (0.53 ~ 3.42)	0.483	0.537
AA						
T1	8 (24.24%)	47 (35.61%)	*r*	*r*		
T2	5 (15.15%)	50 (37.88%)	0.59 (0.18 ~ 1.92)*	0.63 (0.19 ~ 2.09)	0.011	0.451
T3	20 (60.61%)	35 (26.52%)	3.36 (1.33 ~ 8.50)**	4.14 (1.53 ~ 11.23)**	<0.001	0.005
PH						
T1	15 (45.45%)	40 (30.30%)	*r*	*r*		
T2	12 (36.36%)	43 (32.58%)	0.74 (0.31 ~ 1.78)	0.78 (0.29 ~ 2.07)	0.507	0.611
T3	6 (18.18%)	49 (37.12%)	0.33 (0.12 ~ 0.92)*	0.31 (0.08 ~ 1.21)	0.034	0.092
PW						
T1	17 (51.52%)	38 (28.79%)	*r*	*r*		
T2	10 (30.30%)	45 (34.09%)	0.50 (0.20 ~ 1.21)	0.49 (0.19 ~ 1.24)	0.124	0.131
T3	6 (18.18%)	49 (37.12%)	0.27 (0.10 ~ 0.76)*	0.27 (0.09 ~ 0.83)*	0.013	0.023
FHD						
T1	11 (33.33%)	44 (33.33%)	1 (0.39 ~ 2.55)	1.00 (0.40 ~ 2.75)	1	0.914
T2	11 (33.33%)	44 (33.33%)	*r*	*r*		
T3	11 (33.33%)	44 (33.33%)	1 (0.39 ~ 2.55)	1.16 (0.33 ~ 4.09)	1	0.871
FNW						
T1	6 (18.18%)	49 (37.12%)	*r*	*r*		
T2	13 (39.39%)	43 (32.58%)	2.47 (0.86 ~ 7.06)	2.82 (0.95 ~ 8.37)	0.092	0.063
T3	14 (42.42%)	40 (30.30%)	2.86 (1.01 ~ 8.12)*	5.27 (1.50 ~ 18.51)**	0.049	0.010
FHNR						
T1	18 (54.55%)	37 (28.03%)	*r*	*r*		
T2	8 (24.24%)	47 (35.61%)	0.35 (0.14 ~ 0.89)*	0.29 (0.11 ~ 0.77)*	0.028	0.013
T3	7 (21.21%)	48 (36.36%)	0.30 (0.11 ~ 0.79)*	0.24 (0.09 ~ 0.66)**	0.015	0.006

*Note*: Logistic regression was adjusted for age, sex, and body mass index. For SHA and CEA, tertile 2 was used as the reference.

Abbreviations: AA, α angle; CEA, center edge angle; FHD, femoral head diameter; FHNR, the ratio of femoral head diameter divided by femoral neck width; FNW, femoral neck width; NSA, neck shaft angle; OA, osteoarthritis; OR, odds ratio; PH, pelvic height; PW, pelvic width; SHA, sharp angle; SA, sourcil angle; T, tertile.

* *p* < 0.05.

** *p* < 0.01.

Among all indicators, the AA and SA had the highest ORs. Compared to that in the control group, the risk of HOA increased by 4.14 times with a larger AA (T3 OR: 4.14 95% CI: 1.53–11.23 *p* = 0.005) and by 4.89 times with a larger SA (T3 OR: 4.89 95% CI: 1.66–14.42 *p* = 0.004).

### PRC

The complete model incorporating age, sex, height, weight, BMI, and anatomical parameters yielded an AUC of 0.887 (95% CI: 0.821–0.953), with the 10 radiographic parameters contributing 71.059% and general population characteristics contributing less significantly (2.067%). Among all the radiographic parameters, the SA showed the greatest predictive contribution (PRC = 13.695%). The detailed data can be found in Table 5.

**Table 5:** AUC and PRC of multivariate models.

	AUC	95% CI	PRC (%)
Full model	0.887	0.821–0.953	100.000
Partial model without other risk factors	0.879	0.812–0.946	2.067
Partial model without radiographic parameters	0.612	0.514–0.709	71.059
Partial model without SA	0.834	0.754–0.914	13.695
Partial model without SHA	0.881	0.816–0.945	1.550
Partial model without NSA	0.881	0.813–0.949	1.550
Partial model without AA	0.882	0.815–0.948	1.292
Partial model without CEA	0.884	0.819–0.949	0.775
Partial model without FNW	0.885	0.818–0.953	0.517
Partial model without FHD	0.886	0.819–0.953	0.258
Partial model without FHNR	0.886	0.819–0.954	0.258
Partial model without PH	0.887	0.820–0.953	0.000
Partial model without PW	0.889	0.826–0.952	−0.517

*Note*: The full model included other risk factors and radiographic parameters. Other risk factors included age, sex, weight, height, and body mass index. Radiographic parameters included SHA, CEA, SA, NSA, AA, PH, PW, FHD, FNW, FHNR.

Abbreviations: AA, α angle; AUC, areas under the curve; CEA, center edge angle; FHD, femoral head diameter; FHNR, the ratio of femoral head diameter divided by femoral neck width; FNW, femoral neck width; NSA, neck shaft angle; OA, osteoarthritis; PH, pelvic height; PRC, proportional risk contribution; PW, pelvic width; SA, sourcil angle; SHA, sharp angle.

## Discussion

### Risk and Protective Factors for HOA

This clinical study explored the impact of radiographic parameters on the occurrence of HOA in the Chinese population based on pelvic X‐ray images. We assumed that the measurement indicators of interest were symmetrical bilaterally and used the anatomical morphology of the healthy side to represent the state preceding the onset of HOA. Statistical analysis of data from the control group confirmed our hypothesis, which is consistent with the results of several previous large‐scale studies^7^, ^9^, ^13^; additionally, several studies have also supported the idea that the healthy side in patients with unilateral HOA can predict the risk of total hip replacement.^17^ By observing and measuring these indicators, we can statistically analyze and predict the future risk of HOA, which is of great significance for the early diagnosis of this disease.

The SA, also known as the Tönnis angle or acetabular roof obliquity, represents the area of bone condensation beneath the acetabular roof cartilage, which is a reaction to stress caused by pressure acting on the iliac joint portion. Generally, a normal SA is 0–10°. An SA greater than 10° implies hip joint instability and developmental dysplasia, while an SA less than 0° increases the risk of pincer‐type femoroacetabular impingement (FAI). A larger SA that is deviated from the horizontal plane can have adverse effects on the distribution of forces within the hip joint, resulting in reduced coverage of the femoral head by the acetabulum and increased unit force per surface area.^9^

For the evaluation of patients with developmental dysplasia of the hip, most scholars recommend using the CEA, SA, SHA, and acetabular depth. Our study revealed that a larger SA is associated with the development of HOA, but the CEA did not show a significant association with the disease. The reason might be that our patient sample was small and we excluded patients with acetabular dysplasia with CEA < 20°. So even though we observed that the center edge angle was not statistically significant, it does not mean that it is not important. In our study, the OR for HOA with a larger SA reached 4.89, and the PRC for a larger SA was the highest, reaching 13.695%. Previous research has indicated that the SA is more strongly associated with the progression of HOA compared to other indices, even the CEA.^18^ But as explained above, our study cannot be used to prove whether SA or CEA is more important because of the number of patients and the CEA cutoff value included in this study.

Many studies have shown that cam deformity may be a risk factor for primary HOA.^6^, ^19^, ^20^, ^21^ In our study, we found that a large AA is a high risk factor for the occurrence of HOA (OR = 4.14, 95% CI: 1.53–11.23, *p* = 0.005), which is consistent with previous research results. This is due to frequent collisions at the junction of the femoral head and neck and the lateral edge of the acetabulum, resulting in continuous shearing force on the acetabular cartilage, leading to premature degeneration of the hip joint and ultimately causing HOA.^22^ Faber et al.^19^ observed the relationship between cam deformity and osteophyte distribution in HOA in the UK Biobank study and found that cam morphology was more strongly related to osteophytes below the femoral head than osteophytes on the upper outer side of the femoral head and acetabulum, indicating that more attention is needed to study the impact mechanism below the femoral head to determine the relationship between cam morphology and HOA. We found that a lower FHNR is also a risk factor for HOA. Doherty et al.^7^ reported that an FHNR lower than 1.27 can indicate a stalk‐like deformity when the hip joint periphery is deformed and is an important risk factor for HOA. Our finding is consistent with their research results, and the specific degeneration mechanism should be closely related to FAI.

Previous studies have indicated a correlation between a reduced NSA and an increased prevalence of HOA.^7^ An observation using cadaveric models also suggested that within the physiological range, an increase in the NSA slows the degeneration of the hip joint.^23^ Other studies have shown that a smaller NSA is a risk factor for HOA, while a larger NSA is associated with morphological changes resulting from HOA. In our study, no significant association was found between an abnormal NSA and HOA, with ORs of 0.97 and 1.34, indicating no statistical significance. Specific mechanisms need to be investigated in larger studies in the future.

PH and PW represent the basic morphology of the pelvis, and a relatively small PH and PW indicate abnormal morphology. In previous studies, the rotational center of the hip joint has often been determined using formulas based on PH,^24^ while others have improved upon this method^25^ to obtain more accurate central positioning. A study of hip joints in Japanese women revealed correlations between a smaller PW and various markers of hip joint dysplasia, with females having a PW less than 26.5 cm being at a significantly greater risk of HOA.^26^ In our study, a smaller PH and PW were identified as risk factors for HOA, possibly because they affect the position of the rotational center, leading to muscle atrophy and weakness around the hip joint, resulting in the onset of HOA. An increased FNW and a lower FHNR imply a smaller weight‐bearing surface area on the femoral head, thereby increasing stress on the joint.^27^ Additionally, similar to cam deformities, this could cause impingement at the proximal end of the femur, contributing to HOA.

### PRC

Regarding the PRC, the radiographic parameters accounted for 71.059% of the total risk, with an AUC of 0.879, indicating the significant value of local imaging abnormalities in HOA. Among all parameters, the SA showed the largest contribution (PRC = 13.695%), indicating its significance. The specific mechanism had been set out above.

### Limitations and Strengths

This is a very meaningful clinical study that filled the research gap of HOA imaging risk factors in Chinese population. This study focused on several imaging parameters and emphasized the importance of SA. This is important for early HOA screening, diagnosis, prevention, and clinical intervention in the Chinese population.

But it is necessary to acknowledge the limitations of this work. First, this is a case–control study, which cannot be used to infer causality. Second, due to the relatively low prevalence of HOA in China, the majority of patients undergoing total hip arthroplasty in hospitals are primarily those with femoral head necrosis, developmental hip dysplasia or femoral neck fracture. As a result, the number of eligible patients meeting the inclusion criteria was limited. This aspect of the study can be improved later by increasing the number of years of patient inclusion and the number of individuals studied. In addition, this study was based on conventional pelvic X‐ray images, which are the most convenient and cost‐effective imaging data available in routine clinical practice. However, pelvic X‐ray images only provide information on a single plane and are susceptible to the influence of pelvic tilt, as the hip joint is a complex structure. Three‐dimensional imaging techniques such as spiral CT can provide more comprehensive information, and in future studies, more sophisticated imaging methods or additional lateral hip X‐ray images can be incorporated. Finally, we only collected demographic factors such as sex, age, height, and weight as confounding factors and did not adjust for known risk factors for HOA, such as physical activity level and occupation. A broader range of measurement indices could be included in future studies, such as the AD, offset value, and newly discovered factors such as the teardrop distance^28^ and the size of the greater and lesser trochanters,^21^, ^29^ to enrich our research.

## Conclusion

Our research revealed that several radiographic parameters in X‐ray images correlate with the incidence of HOA. Larger SA, lager AA, wider femoral neck, smaller FNHR, and smaller PW were risk factors for HOA. The SA contributed the most and can be used in screening populations at risk for HOA.

## Funding

This work was supported by Fund Program for the Scientific Activities of Selected Returned Overseas Professionals in Shanxi Province (20210006).

## Conflict of Interest Statement

The authors declare that they have no competing interests.

## Ethics Approval

Our study was conducted with the approval of the Medical Research Ethics Committee of the Second Hospital of Shanxi Medical University.

## Authors Contributions

Fan Wang: methodology, validation, formal analysis, writing—original draft, writing—review and editing. Peng Yuan: investigation, data curation, writing—review and editing. Yixin Gong: data curation, visualization, writing—review and editing. Guohao Zhang: visualization, writing—review and editing. Pengcui Li: writing—review and editing. Qiang Jiao: conceptualization, supervision, project administration, funding acquisition.
